# ‘Hairy honours of their chins’: whiskers and masculinity in early nineteenth-century Britain

**DOI:** 10.1080/03071022.2022.2112863

**Published:** 2022-10-06

**Authors:** Alun Withey

**Affiliations:** University of Exeter

**Keywords:** Whiskers, facial hair, masculinity, gender, sexuality, nineteenth century, embodiment, self-fashioning, consumption

## Abstract

Studies of the Victorian ‘beard movement’ of the 1850s have demonstrated the close connections between facial hair and shifting ideas of, and concerns about, masculinity, gender, sexuality and modernity. The ‘beard movement’ is generally seen as the return of facial hair after 150 years of beardlessness. The turn of the nineteenth century, however, witnessed a new and previously overlooked fashion for side-whiskers among young British men, one that initially caused controversy and ridicule, but which gradually became acceptable as a male accoutrement, and spurred a market for cosmetic products. What might be termed the ‘whiskers movement’ of the early 1800s offers a new and earlier perspective on facial hair as a form of embodied masculinity, and its place in contemporary debates about manliness, male fashion and appearance, sexuality and effeminacy, and political and revolutionary affiliations.

In 1843, a tongue-in-cheek article appeared in the New Orleans *Picayune* newspaper, titled ‘Whiskers. Or, a clean shave’. Dwelling on their utility as ‘ornamental appendages to the human face’, the authors sought to discuss how whiskers contributed to the ‘“masculineness” of manhood’, and sought what they considered to be the long overdue return of facial hair. Whereas moustaches and beards were dismissed as ‘an unerring indication of a lack of brains’, whiskers betokened positive qualities, such as honesty, firmness and good nature. It was even suggested that a new branch of natural sciences should be dedicated to their study – ‘*Whiskerology*’.^1^^1^D. Corcoran et al., *Pickings from the Portfolio of the Reporter of the New Orleans ‘Picayune’* (Philadelphia, 1843), 160. Had they but known it, their article was timely. A mere few years later, the moustache and then the beard would indeed make a spectacular return to popularity, overturning a preference for the shaved face that has long been assumed to have lasted for 150 years. In fact, however, the first decades of the 1800s had already seen an emerging trend for whiskers on the faces of British men. Although nothing like as popular or widespread as the later Victorian beard trend, the reappearance of hair on the faces of young British men created tensions and opened up new debates about what was acceptable in terms of male appearance.^2^^2^For the social importance of shaving and the smooth face in the eighteenth century see Alun Withey, ‘Shaving and masculinity in eighteenth-century Britain’, *Journal for Eighteenth-Century Studies*, 36, 2 (2013), 225–43.

Studies of masculinity have frequently been concerned with questions of male hegemony, norms and ideals. The concept of ‘hegemonic masculinity’ emerged in the 1980s, from Raewyn Connell’s ‘social theory of gender’ and discussions of power relationships between men and women.^3^^3^See R.W. Connell, *Gender and Power: Society, the person and sexual politics* (Oxford, 1987). Here, gender behaviours were conceived as being enacted through social practice – what people actually ‘do’ rather than what is prescribed or expected.^4^^4^For a useful summary and critique of the concept see Demetriakos Z. Demetriou, ‘Connell’s concept of hegemonic masculinity: a critique’, *Theory and Society*, 30, 3 (2001), 337–61. As Connell and James Messerschmidt have noted, early studies of ‘hegemonic masculinity’ in the 1980s and 1990s proposed a framework of understanding that saw certain (often minority) patterns or behaviours in the past defined as normative, reflecting or establishing ideals of masculinity.^5^^5^R.W. Connell and J. Messerschmidt, ‘Hegemonic masculinity: rethinking the concept’, *Gender and Society*, 19, 6 (2005), 832. They also note the ‘conceptual confusion’ emerging not only from the sheer range of approaches to the topic, but also the existence of multiple versions of masculinities at any given point.^6^^6^*ibid*., 836. Connell’s model has been criticised for misunderstanding the relationship between hegemonic and non-hegemonic models, and their formation.^7^^7^Demetriou, *op. cit*., 346–47. More recently, as Joanne Begiato has argued in her comprehensive survey of the historiography of masculinity, the construction of masculine identities in the long nineteenth century encompassed a complex mix of body, emotion and material culture, and there are problems in assuming hegemony in terms of either ideals of male appearance or their application.^8^^8^J. Begiato, *Manliness in Britain: Bodies, emotion and material culture* (Manchester, 2020) 3–4.

Attempting to fit masculinity into neat and linear chronological compartments is useful in terms of assessing broad changes, but equally problematic in imposing an order or uniformity that did not necessarily exist on the ground.^9^^9^*ibid*., 4. Equally, by the very nature of the sources used in assessing them, many assumptions about male corporeal ideals are naturally weighted towards middling and elite men. The recent ‘somatic turn’ in the history of masculinity has seen the emergence of a wealth of studies focusing particularly on the form, appearance and features of the male body, with the eighteenth and nineteenth centuries being particularly well served.^10^^10^R. Cooter, ‘The turn of the body’ in R. Cooter and C. Stein (eds), *Writing History in the Age of Biomedicine* (Yale, 2013), 91–111. These have ranged from broad discussions of masculine ideals and body ‘types’ to micro analyses of individual features, areas or surfaces of the male body.^11^^11^For general studies of the male body see D.M. Turner, ‘The body beautiful’ in C. Reeves (ed.), *A Cultural History of the Human Body in the Age of Empire* (London, 2010), 113–32; M. Hau, ‘The normal, the ideal and the beautiful: perfect bodies during the age of empire’ in M. Sappol and S.P. Rice (eds), *A Cultural History of the Human Body in the Age of Empire* (London, 2010), 149–70; and Begiato, *Manliness in Britain*, *op. cit*. Part of this has been a new focus upon the rugged, fit bodies of fighting men and manual workers, and the toned bodies of athletes, which at different points provided ready models of physicality for civilian men.^12^^12^The literature is now extensive, but for some examples see J. Begiato, ‘Between poise and power: embodied manliness in eighteenth- and nineteenth-century British culture’, *Transactions of the Royal Historical Society*, 26 (2016) 125–47; M. McCormack, *Embodying the Militia in Georgian England* (Oxford, 2015); J. Bourke, *Dismembering the Male: Men’s bodies, Britain and the Great War* (Chicago, 1996); A. McIvor and R. Johnston, ‘Dangerous work, hard men and broken bodies: masculinity in the Clydeside heavy industries’, *Labour History Review*, 69 (2004), 135–52; D.E. Hall (ed.), *Muscular Christianity: Embodying the Victorian Age* (Cambridge, 2006 edition). As well as bodily ideals, several recent studies have focused on the ‘othering’ of certain groups of male bodies. Kathleen M. Brown, for example, has explored understandings of black physicality and masculinity in early America (Figure 3).^13^^13^K.M. Brown, ‘Strength of the lion … arms like polished iron’ in Thomas A. Foster (ed.), *New Men: Manliness in Early America* (New York and London, 2011), 172–92. Other essays in this collection also explore the importance of the corporeal bodies of both European and American men. David Turner has revealed the complex meanings attached to impaired bodies in the eighteenth and nineteenth centuries, and the stigmatisation of ‘deviance’ in bodily appearance.^14^^14^D.M. Turner, *Disability in Eighteenth-Century England: Imagining physical impairment* (London, 2012). The place of the queer body as a masculine ‘other’ has also been explored in both periods. One of the most significant developments, however, has been the growing focus on what has been termed ‘embodied manliness’ – the centrality of the body as both a site of, and vector for, prevailing ideas about masculinity. As Karen Harvey has argued in her study of the symbolic importance of the male leg in the eighteenth century, masculinity could be embodied as well as performative, with individual parts of the male body both carrying and conveying meaning.^15^^15^K. Harvey, ‘Men of parts: masculine embodiment and the male leg in eighteenth-century England’, *Journal of British Studies*, 54, 4 (2015), 799.

One feature of the male body that has proved a useful exemplar of embodied manliness in recent historiography is facial hair. As well as being a key marker of masculinity, at certain points it has also been regarded as a bodily ‘other’. Studies of beards in the early modern period, such as Eleanor Rycroft’s recent survey of depictions of beards on the early modern stage, have revealed the complex range of meanings with which it was freighted.^16^^16^E. Rycroft, *Facial Hair and the Performance of Early Modern Masculinity* (London, 2019). On the one hand beard-wearing could suggest vanity, low morals or bodily weakness. On the other, however, as Mark Albert Johnston has shown, beards could signal strength, virility, mental acuity and health.^17^^17^M.A. Johnston, *Beard Fetish in Early Modern England: Sex, gender, and registers of value* (Farnham, 2011), 43–46. Attempts to ‘fix’ the nature and status of the beard saw its establishment as both a proxy phallus, and a broader synecdoche for the male body.^18^^18^*ibid*., 49. For Will Fisher early modern beards were markers of masculine identity, that both constituted and reflected manliness. They were, as Fisher argues, ‘a component of manhood [and] a means through which manhood was materialized’.^19^^19^W. Fisher, *Materialising Gender in Early Modern English Literature and Culture* (Cambridge, 2006), 99. In the eighteenth century, however, shifting attitudes towards facial hair reflected broader changes in ideas about ‘polite’ male appearance.^20^^20^Withey, ‘Shaving and masculinity’, *op. cit*., 225–43. By 1700, displaying facial hair had become increasingly unfashionable, and even to some extent stigmatised. The clean-shaven face was now generally the male standard in Europe. Both the act of shaving and the smooth, open countenance suggested refinement and elegance, as well as exemplifying authority and control over the body. Even so, the ability to grow a beard was still signally important, demonstrating the continuing centrality of facial hair as a key totem of the male body.^21^^21^*ibid*.

Studies of facial hair in the nineteenth century are dominated by the so-called ‘beard movement’. The reappearance of beards in the early 1850s occurred during a period when masculinity was being challenged and remade.^22^^22^For these changes see Begiato, *Manliness in Britain*, *op. cit*., ch. 1, esp. 5–10; J. Tosh, ‘Masculinities in an industrial society, 1800–1914’, *Journal of British Studies*, 44, 2 (2005), 330–42; J. Tosh, *Manliness and Masculinities in Nineteenth-Century Britain* (London, 2005), ch. 3. As greater attention began to be paid to the attributes and physicality of the male body, the beard was repurposed as a key indicator of masculine traits such as strength and character. As Christopher Oldstone-Moore and others have argued, new ideas about the beard mapped onto shifting theories about men and their bodies, and also perceived threats to traditional patriarchal authority.^23^^23^C. Oldstone-Moore, ‘The beard movement in Victorian Britain’, *Victorian Studies*, 48, 1 (2005), 8; S. Walton, ‘From squalid impropriety to manly respectability: the revival of beards, moustaches and martial values in the 1850s in England’, *Nineteenth-Century Contexts*, 30, 3 (2008), 229–45; J.H. Rumsby, ‘Of no small importance’: a social history of the cavalry moustache c.1790–1860’, *Journal of the Society for Army Historical Research*, 96, 386 (2018), 152–67. These included the physical and emotional challenges faced by men of adapting to a newly industrialised society, the increasing unease surrounding masculine authority – and its exercise both in the workplace and the home – and fears about effeminacy and perceptions of the physical and moral laxity of the male population in the mid-century.^24^^24^J. Middelton, ‘The beard and Victorian ideas of masculinity’ in D. Janes (ed.), *Back to the Future of the Body* (Cambridge, 2007), 30–34. Amid such pressures, the beard took on new importance as a symbol of ‘natural’ and timeless male authority and strength. A raft of popular and pseudo-medical literature emerged from the early 1850s both supporting and perpetuating the supposed benefits of beard-wearing.^25^^25^For a few examples see ‘The beard movement’, *The Leader*, 10 December 1853, 1183; ‘A constant reader’ and ‘Beards and moustaches’, *The Daily News*, 2 December 1853, 4; Anon, ‘Sanitary view of the beard and moustache’, *Daily News*, 12 August 1853, 2; Anon, ‘Philosophy of beards’, *Ipswich Mechanics Institution*, 25 March 1854, 2; Anon, ‘Beard and moustache movement’, *Sheffield Independent*, 24 December 1853, 4.

Nevertheless, it could be argued that the criticisms of class and periodisation noted by Begiato could certainly be levelled against the historiography of facial hair which, perhaps most obviously after 1700, tends to follow accepted ‘types’ (the ‘man of feeling’ or the ‘muscular Christian’, etc) and is strongly linked to middling and elite men.^26^^26^Begiato, *Manliness in Britain, op. cit,* 5. As I have argued elsewhere, assumptions that men across society in these periods held the same views about facial hair, or adopted the same fashions, are problematic.^27^^27^A. Withey, *Concerning Beards: Facial Hair, Health and Practice in England, 1650–1900* (London, 2021), 163–64. In the eighteenth century, for example, ‘wanted’ advertisements for runaway servants, apprentices or criminals (presumably lower down the social scale) frequently noted the presence of facial hair in physical descriptions at a time when it has been assumed that all men were clean-shaven.^28^^28^*ibid*., 170–73. Analysis of hundreds of prisoner photographs in the nineteenth century likewise suggests different patterns in the wearing and style of facial hair according to various factors such as age, location and status.^29^^29^*ibid*., 176–84. Such variations further reveal the dangers of assuming hegemonic masculinity in any given period, and add weight to the argument that, rather than single masculine types that were uniformly understood and applied across respective societies, men could potentially be part of several overlapping versions of masculinity and manliness according to many factors including class, location, occupation and sexual identity.^30^^30^A point made by Joanne Begiato, who notes the unreliability of masculine identity as a fixed, or uniform experience – Begiato, *Manliness in Britain, op. cit*., 6–7. As such, alternative versions of embodied manliness could emerge *within* prevailing masculine ideals or types (rather than necessarily in opposition to them), and could be culturally or temporally delimited.

The emergence around 1800 of a fashion for one particular style of facial hair – whiskers – however, offers a useful means of interrogating such alternative or what might perhaps be termed ‘micro’ masculinities. Whiskers have so far received relatively little attention in the literature, and within the existing narrative of facial hair fashions, are usually a sidenote. One notable exception is Maria Alonso’s study of the revolutionary symbolism of whiskers in nineteenth-century Spain, exploring their importance as a visual and corporeal shorthand for radical political affiliation.^31^^31^M.V. Alonso, ‘Beardless young men? facial hair and the construction of masculinity in nineteenth-century Spanish self-portraits’ in J. Evans and A. Withey (eds), *New Perspectives on the History of Facial Hair* (London, 2018), 91–108. M.C. Newbould has also explored literary references to, and meanings of, whiskers in poetry, and in the context of culture and identity among Cambridge undergraduates in the 1830s.^32^^32^M.C. Newbould, ‘*The Rape of the Whisker* and *Fuzzwhiskiana*: Regrooming Pope’s *Rape of the Lock* in early nineteenth-century Cambridge’, *Philological Quarterly*, 95, 1 (2016), 125–48. Aside from these studies, little attention has focused on this style of facial hair and its potential for nuancing arguments about manly appearance and masculine identities.

This article explores the fashion for whiskers in Britain in the first decades of the nineteenth century, a version of embodied manliness that was specifically linked to young, predominantly urban and elite men, and was also both temporally and geographically limited. This fashion ran counter to the generally accepted model of appearance from the late eighteenth century, where facial hair was assumed to be in opposition to ideals of neatness and elegance. The return of first moustaches, then beards, from the early 1850s is generally set against nearly a century and a half of beardlessness. What might be termed the ‘whisker movement’ of the early nineteenth century, however, both challenges this assumption, and offers a new interpretation of the nature and form of debates surrounding the return of facial hair.

The article advances several arguments. First, it offers further evidence of the place of facial hair within concepts of embodied masculinity. Except for some work on moustaches, many discussions of facial hair fashions and masculinity are based around a simple binary between periods of preference for the bearded or non-bearded male face. This article suggests that greater attention to specific styles reveals the complexities of the relationship between facial hair and masculine identity, as well as highlighting the dangers of applying neat chronological frameworks, without considering local and temporal variations. Rather than simply showing how individual characteristics of the body informed broader and prevailing masculine identity, it instead turns this formulation on its head and explores how a particular style of facial hair contributed to a version of masculinity that emerged both *within*, and to some extent in opposition to, the generally accepted model. There is evidence, for example, that whisker-wearing was ‘othered’, and was initially regarded as potentially effeminising. Equally, there are questions about the extent of the fashion, across age, class and location. As such this article provides further caution of the dangers in assuming hegemony in masculine ‘types’ and normative male appearance.

Second it challenges the assumption that the mid-nineteenth century was necessarily even a key point of change in facial hair fashions, arguing instead that the return of facial hair began decades earlier. Many arguments made in defence of whiskers neatly prefigure those made about the beard in the 1850s and 1860s, including physical and moral strength, martial models of masculinity, appeals to bearded heroes in antiquity, and remarkably similar claims as to their innate manliness and place as a natural adornment. Equally, as occurred during the ‘beard movement’ in the 1850s, the popularity of whiskers was supported by a growing range of cosmetic products, even spawning an apparent fashion for imitation by women. There were also marked similarities in the contexts of the return of whiskers and, later, beards. As did arguments about beards in the 1850s, early debates about whisker-wearing in the early 1800s focused upon factors such as national identity (and xenophobic attitudes towards ‘foreign’ bodies), gender and effeminacy, and also the place of facial hair as an adornment of the soldier. Again, as occurred during the 1850s, the fashion for whiskers also took place against the backdrop of military and political tensions in Britain, and a deeper sense of unease about masculinity and manliness. The emerging fashion for whiskers therefore suggests that debates about the nature and status of facial hair and the male body potentially emerged much earlier than the mid-nineteenth century, and as such reflect deeper and longer concerns about male corporeality and manly appearance.

The article begins with a discussion of both the terminologies of ‘whiskers’ and the trajectory of the new trend before turning, in the second section, to contemporary reports, debates and responses to this new phenomenon. As will be shown, such debates cut across a variety of ideas, such as gender and sexuality, including suspicions of effeminacy among whisker-wearers, but also the place of facial hair in embodying martial masculinity. The discussion also explores the place of facial hair within concepts of corporeality, race and nationhood, and its significance as a cultural marker. The final part of the article further explores depictions of whiskers in popular culture and satire, particularly the supposed connections between whiskers and ‘dandyism’. It also argues, however, that, despite such pejorative attitudes, the fashion was clearly significant enough to spur the emergence of a market for cosmetic products to style and encourage the growth of facial hair.

## Defining whiskers

The first point to consider, then, is what were ‘whiskers’ and how were they understood by contemporaries? Matters are complicated by the considerable degree of slippage in the term, not only in the nineteenth century, but also when deployed by historians. Throughout the long eighteenth century and beyond, the word ‘whiskers’ was interchangeable (indeed, virtually coterminous) with the moustache. Moustaches could be defined *as* whiskers. In 1735, for example, Benjamin Defoe’s *Compleat English Dictionary* defined ‘Mustaches’ as ‘that part of the beard growing on the upper lip; Whiskers’.^33^^33^B. Defoe, *A Compleat English Dictionary: Containing the true meaning of all the words in the English language* (Westminster, 1735), entries in alphabetical order. For Charles James in 1802, a moustache was ‘literally […] the hair growing on the upper lip of a man, and which is better known among us by the familiar term “whiskers”’.^34^^34^C. James, *A New and Enlarged Military Dictionary, or Alphabetical Explanation of Technical Terms* (London, 1802), 5. See also N. Webster, *A Dictionary of the English Language, Compiled for the Use of Common Schools in the United States* (Hartford, CT, 1817), 212, where the entry simply reads ‘Mustaches or Mustachoes, *n, pl*., whiskers’. Likewise, some definitions of the term ‘whiskers’ implied, or even made explicit, that they were referring to moustaches.

**Figure 1.**
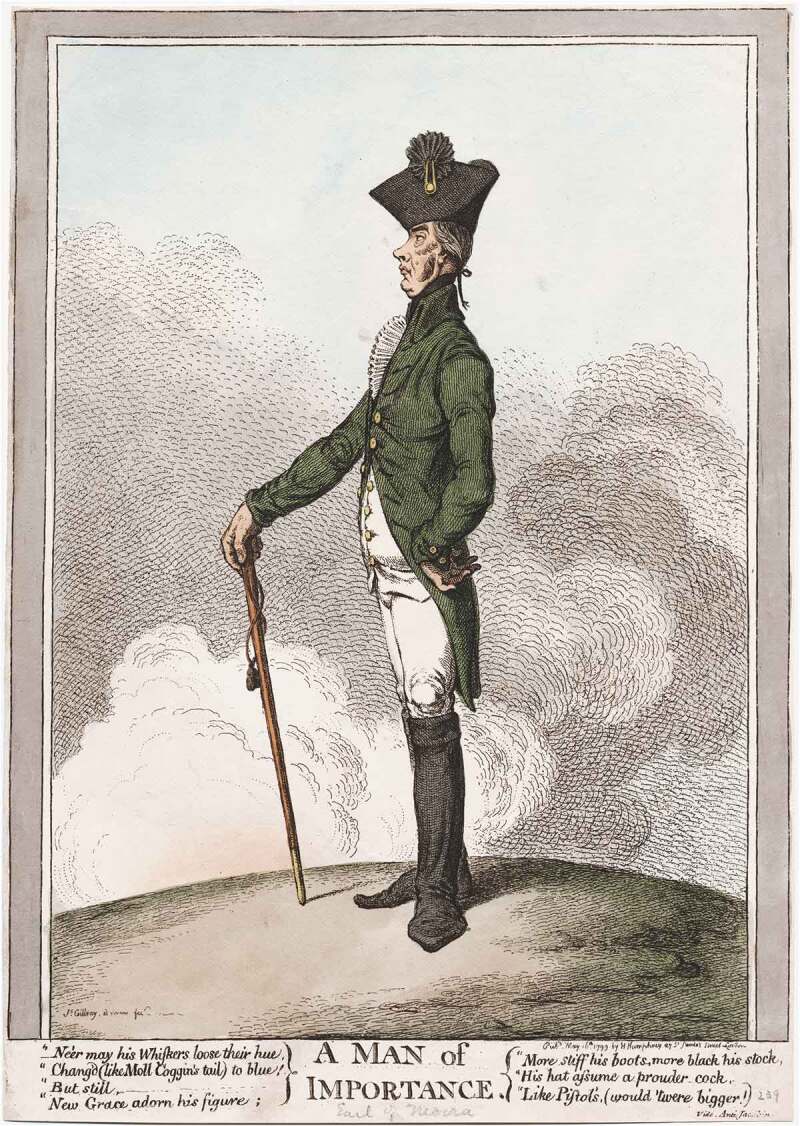
James Gillray, *A Man of Importance* (London: Printed by Hannah Humphrey, 1799). Image courtesy of Lewis Walpole Library, Yale University.

There was also ambiguity, however, since whiskers could equally be understood as outcrops of hair on the sides of the face, growing down from the sideburns. Like moustaches, whiskers came as a pair. James Gillray’s *A Man of Importance* (1799) satirising Lord Moira, the Marquess of Hastings, depicted the Marquess with side-whiskers, and no moustache. In the accompany text, ‘Ne’er may his whiskers lose their hue / Chang’d (like Moll Coggins’ tail) to blue’ (Figure 1).

There is certainly evidence that whiskers could represent, and be understood as, an entirely distinct style to beards and moustaches. In June 1813, in a letter to the editor of a British newspaper, ‘Aenobarbus’ wrote in defence of the ‘growing custom of encouraging whiskers *and* [my emphasis] mustachios’ in Britain. The separation of the two terms here implies that side-whiskers were understood to be separate to moustaches.^35^^35^‘Aenobarbus’, ‘Whiskers and mustachios’ quoted in Anon, *The Spirit of the Public Journals for 1813* (London, 1814), 147. There is also evidence that this definition of whiskers became stricter through the 1810s and 1820s. In 1823, the Suffolk lexicographer Edward Moor suggested that whiskers referred to ‘the hair on the upper lip as until lately, I believe, all over England’, but also noted that the term now also encompassed ‘the hair under the ears, sometimes under the eyes also, bear[s] this term, and the labial comæ, are called moustaches’.^36^^36^E. Moor, *Suffolk Words and Phrases, or an Attempt to Collect the Lingual Localisms of that County* (Woodbridge, J. Loder, 1823) 482. The key phrase was ‘until lately’, suggesting that the term was in the process of being fixed. By 1837, the term ‘whiskers’ had generally come to signify side-whiskers. The character Smangle in Dickens’s *Pickwick Papers*, for example, was referred to as ‘A tall fellow, with … very thick bushy whiskers meeting under his chin’.^37^^37^C. Dickens, *The Posthumous Papers of the Pickwick Club*, 1st book edition (London, 1837), 41. This description is characteristic of large, ‘mutton-chop’ sideburns, or perhaps even the ‘chin curtain’ style, with large side-whiskers but no beard or moustache. In describing whiskers, nineteenth-century authors exhausted virtually their entire store of florid adjectives. They were variously described as ‘labial excrescences’, ‘hairy honours of [men’s] chins’, ‘manly appendages’ and ‘ornaments of manhood’.^38^^38^Speculator, ‘Whiskers!!! And the Baron Geramb’, *The Scourge: Monthly Expositor of Imposture and Folly* (London, 1811), 257; ‘Aenobarbus’, ‘Whiskers and mustachios’, *op. cit*., 147; ‘Rowland’s macassar oil’, *Hampshire Advertiser*, 7 March 1846, 1. The focus of this article will be ‘whiskers’ understood as hair growth on the sides of the face, rather than moustaches.

An emerging fashion for whiskers in France and Germany had begun to be noted in the English press in the early 1800s. By 1806 it was clear that the trend had spread to Britain. In December that year *The Hereford Journal* reported a new trend for ‘enormous whiskers’, to which some men had begun to ‘add Jewish moustachios’, which the writer considered an ‘odious barrier’.^39^^39^Anon, ‘Friday’s post concluded’, *The Hereford Journal*, 3 December 1806, 4. The use of ‘add’ implies that there was some conceptual distance between moustaches and whiskers, and that they were considered separate entities. By 1812 the trend was apparently in full flower, and certainly appears to have been popular in London. Reports of the emerging fashion, however, suggest a mixed reception. Some, such as a correspondent to *The Tradesman or Commercial Magazine* in July 1812, professed astonishment at the ‘spreading proportion of hair on the human face’ he witnessed there, describing it as nothing less than a ‘whiskered *mania* [original italics]’ which had ‘very far over-stepped its bounds’.^40^^40^T. Bobbin Jr, ‘On the absurdity of whiskers’, *The Tradesman or Commercial Magazine*, 9 July 1812, 29–30. Such views echoed earlier associations of facial hair with unfavourable stereotypes, such as the rustic fool, the derelict, the wild asylum patient, the revolutionary or the social dropout.

Women were also initially cautious about the return of facial hair among men. One popular trope was the suggestion that men loved their whiskers more than their partners, and that the former should be removed as a token of true love. Tales such as ‘The whiskers’ in which a Grenadier (‘with the most martial pair of whiskers’ in the whole army) refuses the request of his inamorata to remove his whiskers before she would marry him, were a sign of the mistrust of male vanity by women, and in fact appeared repeatedly between 1806 and 1836 in various publications.^41^^41^See for example ‘The whiskers’, *The Mirror of Literature, Amusement and Instruction*, 31 May 1823, 28–30. See also ‘Eliza’, ‘The value of whiskers’, *The Lady’s Monthly Museum*, 12 March 1812, 152–53. In diary entries, too, women were sometimes less than enthusiastic about whiskered men. Commenting on the appearance of a French minister in 1800, Lady Melesina Trench was unimpressed, noting that the man’s whiskers ‘contributed to the dinginess of his appearance’.^42^^42^R.C. Trench (ed.), *The Remains of the Late Mrs Richard Trench, Being Selections from her Journals, Letters and Other Papers* (London, Parker, 1862), 520. In 1811, Lady Sydney Owenson entreated her husband not to grow his whiskers too long, even including two caricatures of him with whiskers in the current style.^43^^43^S. Owenson, Lady Morgan, *Lady Morgan’s Memoirs, Autobiography and Correspondence, Volume 1* (London, 1862), 528.

Others, however, were more supportive. A correspondent to the *Morning Chronicle* in 1812 was alarmed by premature reports that whiskers were falling out of fashion, having himself ‘been occupied for some time in nourishing a pair of *Levee* [original italics] whiskers’.^44^^44^‘O’, ‘To the editor of the Morning Chronicle’, *The Morning Chronicle*, 14 April 1812. Another cited the whiskers of Confucius as a symbol of wisdom, arguing that such illustrious connections made it no surprise that whiskers made the weak appear strong, the old appear young, the cowardly appear brave, and the ugly look beautiful.^45^^45^‘Whiskers’, *Liverpool Mercury*, 1 January 1813. Perhaps the most spirited defence of whiskers was made by ‘Aenobarbus’, mentioned above. Noting the numerous witticisms lately aimed at whisker-wearers in the British press, he mounted a spirited defence. Whiskers, he argued, conjured up the ‘grave and manly countenance’ of the ancients. They were ‘natural’ and even ‘beautiful’, whereas shaving was a cruel and unnatural act, which disfigured the ‘Human Face Divine’.^46^^46^‘Aenobarbus’, ‘Whiskers and mustachios’, *op. cit*., 147–50. Such arguments neatly prefigure those made at the height of the ‘beard movement’; facial hair was depicted as a natural, God-given, handsome emblem of the male face. In an age ‘so attached to antiquities’, it was ‘silly to oppose so ancient a custom’ as the cultivation of whiskers.^47^^47^*ibid*., 150. After their initial reluctance, women, too, began to embrace the change in male appearance. In 1806, it was reported that the ‘dowagers of the Whiskerando tribe’ were now much in favour of it.^48^^48^Anon, ‘Whiskers’, *The Sporting Magazine*, October 1806, 179.

Assessing how widespread the fashion was, and how far down the social scale it penetrated, is problematic, not least due to the limitations of source materials. Many references occur in newspapers and periodicals, which are necessarily skewed towards metropolitan middle classes and elites, and there is also scant evidence for the motivations of individual men in their choice of facial-hair style. It does seem clear that the trend for whisker-wearing was particularly strong among young, urban elite men in the south of England. Indeed, for some, whiskers were *the* fashionable adornment of the young city *beau*.^49^^49^‘Eliza’, ‘The value of whiskers’, *op. cit*., 153. The popular song ‘The Grand Panorama in London’ lauded the vibrant culture of the capital and its inhabitants, and included the verse ‘Our bucks and gay loungers of spirit and fashion/For whiskers terrific betray a strong passion’.^50^^50^‘New songs’, *The Ladies’ Fashionable Repository*, Date unknown – c.1810, 26. Elsewhere whiskers were noted as being popular among ‘our young bucks of distinction’.^51^^51^Anon, ‘Friday’s post concluded’, *The Hereford Journal*, 3 December 1806, 4. The place of whiskers as a fashionable adornment for young elite men is reinforced by Charles Tindal’s young protagonist – a Cambridge undergraduate – in his poem *The Rape of the Whisker*, whose own whiskers (‘once, alas! the boast of Trinity, on which their owner doated to infinity’) were shaved off by a rival after a night’s drinking.^52^^52^Quoted in Newbould, ‘*The Rape of the Whisker*’, *op. cit*., 129.

There are also suggestions of the spread of the trend to other levels of society. The diary of an imagined apprentice in ‘The Scourge’, for example, noted that ‘If my whiskers don’t grow soon I’ll buy a pair of false ones, *for whiskers I must have*’ [my emphasis].^53^^53^Anon, ‘The London Apprentice’s Journal; or how to pass a Sunday’, *The Scourge or Literary, Theatrical and Miscellaneous Magazine*, 7 February 1814, 106–07. The suggestion, although humorously intended, was that whiskers had become an essential accoutrement for any young man with social pretensions. Anecdotal evidence from court records and ‘wanted’ criminals suggests that men lower down the social scale were wearing whiskers. At a time when being clean-shaven was still likely the broad standard in male appearance, whiskers could prove a useful (if unreliable due to their prosthetic nature) distinguishing feature through which criminals and runaways might be identified. Thus in 1810, a man attempting to defraud women by impersonating a sheriff’s officer was described as being 5 feet 8 inches tall, with a sallow complexion ‘and huge whiskers’.^54^^54^‘Accidents, offences etc’, *The Examiner*, 11 November 1810, 720. For other examples see the report of the swindler ‘Harris’, in *The Norfolk Chronicle, or Norfolk Gazette*, 2 September 1809, 2; ‘Court of Kings Bench’, *The Examiner*, 25 February 1810; ‘Postscript’, *The Lancaster Gazette*, 4 January 1812. There are obvious problems, however, in assuming that the underlying motivations for wearing whiskers were consistent across the social spectrum, or that they reflected the same version of manliness.

By no means either was this a fleeting trend. In 1818, a (presumably imaginary) letter from a London lady to her sister recommended that her uncle ‘continue his handsome whiskers’ but should shave off his moustache before visiting London, suggesting that the trend was still popular.^55^^55^‘Letter from a lady in London to her sister in the country’, *La Belle Assemblée, or, Bell’s Court and Fashionable Magazine*, 1 June 1818. Susan Walton notes that side-whiskers were one of the only facial-hair styles to be tolerated in Britain before 1850.^56^^56^Walton, ‘From squalid impropriety’, *op. cit*., 229. By 1830, though, the fashion had begun to stall. An article in *The Age* in 1830 bemoaned the apparent demise of whiskers and quoted ‘Madame Du Maurier’ in asking what had become of ‘all the gold that used to brush [young men’s] bosoms?’^57^^57^Anonymous, ‘Easy shaving’, *The Age*, 4 April 1830.
*The Morning Post* in June the same year seemed less nostalgic, arguing that while ‘some dandies have tried to bring in the fashion of tufts of frizzed hair on each side of the face’, the only acceptable facial hair for a true gentleman of fashion was to have a beard only on his chin.^58^^58^Anon, ‘Gentleman’s fashions’, *The Morning Post*, 30 June 1830.

That there was a potentially substantial and widespread fashion for whiskers in the early decades of the nineteenth century, then, appears clear. The question remains as to why it occurred then, and what the debates that surrounded the return of whiskers can reveal about manliness and the male body. As will be shown, the fashion for whiskers can be linked to a number of factors in the early nineteenth century, including identity, gender and sexuality, the rise of xenophobic attitudes towards foreign ‘others’, and complex and sometimes contradictory attitudes towards fighting men as models of masculinity.

### The whiskers debate (1) – martial and ‘foreign’ masculinities

If, as Oldstone-Moore argues, the ‘beard movement’ was partly an expression of ‘muscular Christianity’, then in what context can whiskers be understood?^59^^59^Oldstone-Moore, ‘Beard movement’, *op. cit*., 9. What had prompted their return after virtually 120 years of the clean-shaven standard? It seems clear that whiskers were closely bound up with broader and deeper changes in conceptions of the male body and masculinity. Like the mid-century ‘beard movement’, the re-emergence of facial hair around 1800 occurred against the backdrop of concerns about masculinity and manly bodies. As Joanne Begiato has noted, the period between roughly 1790 and 1850 was one of transition in terms of the idealised appearance of the male body. It was ‘becoming solid, broader, rugged and perhaps less elegant’.^60^^60^Begiato, ‘Between poise and power’, *op. cit*., 133. Such changes took place amid a growing sense of national crisis, due to the Revolutionary and Napoleonic Wars, and concerns about the fitness of a thin or fey male body to adequately perform in combat.^61^^61^*ibid*., 134. As it did later in the century, the military body offered civilian men an ideal of heroic manliness, and facial hair was deeply bound up with these associations.^62^^62^See Oldstone-Moore, ‘Beard movement’, *op. cit*., 12–14.

The imitation of military heroes as an explanation for the reappearance of whiskers appears, at first, unlikely, given that the majority of British regiments were clean-shaven in the early nineteenth century. In some British regiments, however, there were certainly prototypes. Some, including the Light Dragoons and Hussars, were notable for their facial hair. These were cavalry regiments, noted for their heroism in battle, leading charges against enemy lines. In 1802, it was reported that the Prince’s Regiment in Brighton had just received orders to ‘let their whiskers grow, that being a preparatory measure to the general assumption of the Hussar habit’.^63^^63^Anon, ‘Brighton’, *Morning Post and Gazetteer*, 14 October 1802. An entry for ‘whisker’ in an 1810 military dictionary noted that they were ‘a superfluous appendage’ worn by specific regiments to distinguish the Dragoons and Hussars from all other soldiers in the British Army. For a young man, then, a set of military whiskers could be grown to imply heroism and strength. The fiancé of ‘Eliza’, grew his whiskers ‘after the example of his brother, who is a lieutenant in the Army’, and told her that he would rather lose his life than his whiskers.^64^^64^‘Eliza’, ‘The value of whiskers’, *op. cit*., 153. This pattern was certainly noted elsewhere in Europe. As Maria Victoria Alonso has noted, a new fashion for whiskers in early 1800s Spain was firmly linked to the desire to emulate military masculinity without the negative associations borne by full beards or moustaches.^65^^65^Alonso, ‘Beardless young men’, *op. cit*., 94–95.

Nevertheless, the wearing of whiskers as a symbolic means of emulating martial masculinity was problematic. Despite the growing estimation of the soldier as an exemplar of manliness, there was an uneasy relationship between civilian men and the martial body. As the example of ‘Eliza’s’ fiancé demonstrates, the desire to look ‘soldierly’ could indeed be a motivation for growing whiskers. The association between facial hair and heroic regiments could be seen as cementing a positive image of whiskers as a desirable adornment. Yet just as soldiers could be lauded, so too could they be mocked for their appearance. Some soldiers even objected to the wearing of moustaches and whiskers, arguing that they were unnecessary, even dangerous. Others pointed to the longstanding tradition of the clean-shaven face of the British soldier, suggesting that this, and not the whiskers or moustache, was the correct form. The author Charles James was clearly no supporter of the habit. Whiskers, he asserted, rendered the soldier ridiculous at home, while not making him any the more ‘terrific abroad’.^66^^66^C. James, *New and Enlarged Military Dictionary in French and English, Volume 2* (London, 1810) – unpaginated, see entries for ‘Uniform’ and ‘Whisker’. For James they were a feature of ‘foreign’ masculinity, and not refined Britishness. Reviewing the uniform of British soldiers in 1809, Sir David Dundas complained about the ‘gaudy trappings’ of ‘whiskers and gold lace’ in military uniforms, which he thought were entirely unnecessary and, worse, smacked of foppery.^67^^67^‘The army’, *The Examiner*, 9 April 1809.

One notorious incident further served to complicate the image of military facial hair in the public’s mind. In July 1806, the removal of whiskers had been an integral element in a large-scale mutiny by Sepoy troops in the Southern Indian town of Vellore. The Sepoy soldiers were part of an Indian army in service to the British crown, as well as in French and Portuguese India. The mutiny revolved around changes to the dress code introduced in May 1806 by the British Commander-in-Chief, Lieutenant-General Sir John Cradock, which included the measures, offensive to Muslim soldiers, of shaving their beards and trimming moustaches.^68^^68^For an account of the mutiny see D. Moodley, ‘Vellore 1806: the meanings of mutiny’ in J. Hathaway (ed.), *Rebellion, Repression, Reinvention: Mutiny in comparative perspective* (Westport, CT, 2001), 89–101; Anon, *The Christian Observer, Conducted by Members of the Established Church for the Year 1813* (Boston, 1814), 239. The order was quickly repealed and supplementary orders issued to allow ‘native troops … to be at liberty to resume their distinctive marks, their ornaments, and their modes of wearing their beards and whiskers’.^69^^69^Anon, *Papers &c (East India Company), Second Part, 24 November 1812–22 July 1813, Volume VIII* (London, 1813), East India Affairs, 7. Nevertheless, the incident drew widespread censure, as well as sympathy for the Sepoy troops, especially from religious groups. While the altering of dress or appearance of European soldiers was of little consequence, the seemingly arbitrary order to remove such a culturally loaded symbol as the Muslim beard was, according to one observer, ‘one of the most wild, extravagant and senseless measures which human folly ever engendered’.^70^^70^Anon, *The Anti-Jacobin Review and True Churchman’s Magazine* (London, 1810), 367. See also ‘The late mutiny in India’, *The Caledonian Mercury*, 19 January 1807. It made little sense to critics that young British soldiers should be cultivating their own whiskers while their commanders were arbitrarily insisting that Indian troops in British service removed theirs.

### The whiskers debate (2) – race and nationality

Race and nationality were in fact key components in broader debates about facial hair. Indeed, the fashion for whiskers reveals important fault lines in terms of both the divergences between British and European ideals of male appearance and, more particularly, xenophobic anti-Gallic and German attitudes in early nineteenth-century Britain. The period following the French Revolution saw an increase in anti-foreign (and, in particular, anti-French) propaganda in Britain. The horrifying spectre of revolution, with all its attendant connotations of madness, chaos and violence, gave rise to a ‘Gallic stereotype’ in British culture, one that ‘drew authenticity and tremendous ideological force’ from the stormy political situation in Europe.^71^^71^G. Newman, ‘Anti-French propaganda and British liberal nationalism in the early nineteenth century’, *Victorian Studies*, 18, 4 (1975), 388. Where ‘Frenchness’ had once exemplified style, fashion and élan, it now came to symbolise all that was undesirable, unsophisticated and un-British. Criticisms of the new trend often stressed ‘foreignness’, suggesting that facial hair belonged to inferior Europeans, and not the refined British male. Early reports, for example, noted the continental fashion for whiskers but were swift to declare it unsuitable. In 1801, the *Ipswich Journal* noted the new French fashion for whiskers, which, it sneered, ‘were spread too far upon the cheek’.^72^^72^Anon, ‘Friday’s post’, *The Ipswich Journal*, 12 December 1801. In January 1802, the *Morning Post and Gazetteer* also noted an emerging trend for facial hair in France but felt confident in asserting that there was little danger of ‘broad and black whiskers’ being imported by British gentlemen. Such ‘disgusting adornments’ were specifically identified as an unwelcome effect of revolution.^73^^73^Anon, ‘Our connections with France’, *Morning Post and Gazetteer*, 2 January 1802. Indeed, they were even held up as a cause: ‘such have been the effects of terror when people let their beards grow’.^74^^74^*ibid*. For the satirical writer ‘Tim Bobbin Jr’, this ‘absurd and indecent fashion’ belonged to Europe; the ‘*visage a la baboon*’ had no place on an Englishman’s face.^75^^75^Bobbin, ‘Absurdity of whiskers’, *op. cit*., 30.

The connection between beards and the distrust of foreign practices, as well as the issue of military belligerence, ran deeper. Being clean-shaven, as Susan Walton notes, was one of the means through which English men signalled their disdain for military conscription, as was mandatory in many European countries. To grow whiskers, then, was potentially to convey pugnacity, which, in turn, could lead to trouble.^76^^76^Walton, ‘From squalid impropriety’, *op. cit*., 233–34. The strength of anti-French feeling, in particular, meant that those displaying conspicuously continental fashions or features risked violence. In 1810, a group of the 15th Light Dragoons were attacked and abused while riding in London, by a mob mistaking them for German soldiers because of their whiskers.^77^^77^James, *New and Enlarged Military Dictionary, Volume 2, op. cit*., unpaginated, see entries for ‘Uniform’ and ‘Whisker’. Even by mid-century, the association between facial hair and rough French or German stereotypes was enough to stir some to action. In 1851, a mere few months before the beard movement took flight, the bearded and moustachioed ‘C.S’. complained to the *Leader and Saturday Analyst* of being mocked and physically attacked on the streets of London for his facial hair. Enduring a hail of stones and gravel, as well as being refused entry to a shop, the final straw for C.S. was being called a ‘French Dog’, not only by ‘common people, and by boys, but also by well-dressed and grown-up people’. The irony, as he pointed out, was that C.S. was actually a veteran of more than 40 years’ experience in the British Army, often guarding against the French.^78^^78^C.S., ‘Moustaches and beards prejudicial to their wearers’, *Leader and Saturday Analyst*, 19 April 1851.

Such was the symbolic strength of whiskers that they could have marked effects on political reputations. Politicians who wore whiskers risked accusations of revolutionary tendencies, or suspect political affiliations. Christopher Oldstone-Moore and Mary Gluck suggest that beards and whiskers were a symbol both of ‘political and cultural radicalism’ and the rejection of bourgeois values in the 1830s and 1840s; in fact such connections were older.^79^^79^Oldstone-Moore, ‘Beard movement’, *op. cit*., 10; M. Gluck, ‘Theorizing the cultural roots of the Bohemian artist’, *Modernism/Modernity*, 7 (2000), 351–78. The Tory politician William ‘Whisker’ Mellish was known for his ‘enormous whiskers’, which, according to political commentators, made him resemble a German, and were regarded as a deliberate sign of his Hanoverian loyalties.^80^^80^Anon, ‘To the free and independent electors city and liberties of Westminster’, *Cobbett’s Weekly Political Register*, 21 March 1807. Mellish was a constant target of political pamphleteers, his whiskers ridiculed as an odd, foreign affectation. Such connections brought to mind uncomfortable connections with Hanoverian troops, stationed in Britain at various points in the late eighteenth and early nineteenth centuries.^81^^81^J. Heinzen, ‘Transnational affinities and invented traditions: the Napoleonic Wars in British and Hanoverian memory, 1815–1915’, *The English Historical Review*, 127, 529 (2012), 1409. Here, again, the link between soldierly appearance and undesirable European stereotypes is telling. It is also worth noting, though, that exotic foreignness could also engender admiration. The visit of Baron de Geramb to London in 1813 caused a sensation, in part because the Baron was possessed of whiskers ‘of unusual size’, which ‘the whole town was in love with’. In Geramb’s case, ‘whiskers’ referred to his almost comically enormous moustaches, which were roundly satirised by pamphleteers. As ‘P.P’. wrote to the *Scourge*, the British were unique among the nations of Europe for their credulity and love of eccentricity.^82^^82^P.P., ‘On the frivolity of the English people’, *The Scourge or Monthly Expositor of Imposture and Folly*, 5 May 1813, 418.

### The whiskers debate (3) – dandyism and sexuality

A third, and perhaps simpler, explanation is that whiskers represented a *fin-de-siècle* reaction against more than a century of a clean-shaven male standard. The timing of the trend may be significant, occurring at a point of transition between, on the one hand, older, eighteenth-century ideas about polite appearance, with shaving regarded as an expression of manly self-control and authority, and, on the other, gradual moves towards a more physically rugged model. In this context it is interesting to revisit the points made by ‘Aenobarbus’ noted earlier, which contained elements of older, Georgian and neoclassicist ideas about the male form, as well as concepts, such as the place of facial hair as a ‘natural’ feature of the male, that would be stressed in the later beard movement. It is possible, for example, given the frequent emphasis upon young men as the vanguard of the new fashion, that the return to a distinctive style of facial hair was a conscious rejection of the patriarchal standards of the older generation, and an attempt to claim a distinctive corporeal aesthetic of their own. This certainly occurred during the later ‘beard movement’, for example, which saw younger men begin to return to smaller beards, moustaches and the clean-shaven face from the 1870s, abandoning the full beards of their fathers and grandfathers.

However, as Newbould argues, the use of whiskers as a particular symbol of masculine identity was problematic. While they were a ‘visible testament of manliness’, their physical resemblance (and semantic proximity) to female pubic hair placed them in a ‘counteractive discourse of suspect masculinity’.^83^^83^Newbould, ‘*The Rape of the Whisker*’, *op. cit*., 141. Partly because of their connections with younger men, but also because the care and attention needed to maintain them bordered on affectation, the fashion for whiskers formed part of wider debates about gender, effeminacy and the ‘dandy’. Concerns about the physical appearance of young men also reflected broader fears about their physical and moral degeneracy. While there was no explicit suggestion that whiskers were an effeminate adornment *per se*, they were seemingly part of the recognised ‘uniform’ of the dandy. The sale of ‘whisker wigs’, noted earlier, came in for a specific attack in the same newspaper in which their maker had advertised them. Noting that the items were coming into fashion, an article criticised the maker for encouraging the ‘smock faced goats who play their wanton tricks through Bond Street’.^84^^84^‘The fashionable world’, *Morning Post*, 9 January 1801, 2. The synonymous association between whiskers and the dandy appears to have been particularly strong after 1815. ‘That kind of man with whiskers large and hair that’s rather sandy/A stiff cravat, gold chain and glass, is what they call a Dandy’, ran a humorous poem in 1822.^85^^85^Anon, ‘The Rout’, *The Mirror of Literature, Amusement and Instruction*, 16 November 1822, 41. One article suggested that dandies hated the current hot weather, since it ‘prevents the whiskers sticking to their cheeks’ and ‘makes their stays uncomfortable’.^86^^86^Anon, ‘Newspaper chat’, *The Examiner*, 23 June 1822, 397. The joke hinged upon the suggestion of affectation, both in the adoption of false whiskers, and the use of corsets to give the illusion of good form.

**Figure 2.**
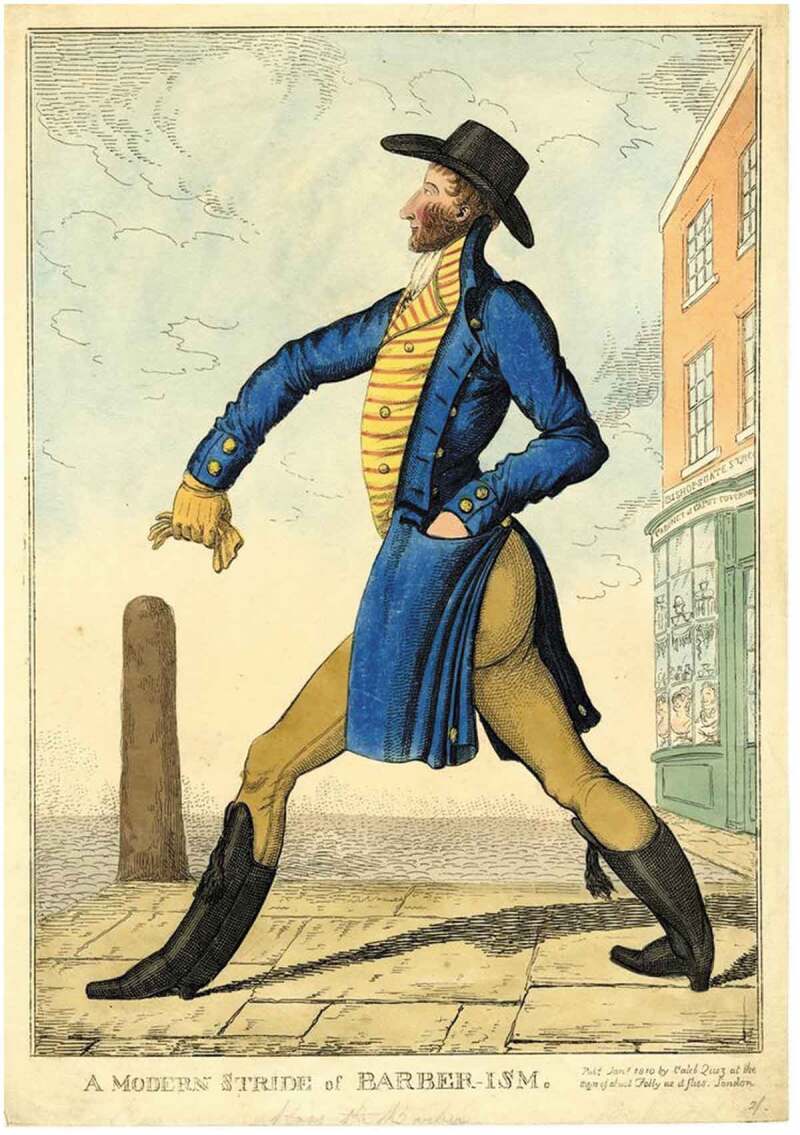
*A Modern Stride of Barber-ism* (1810). *©*The Trustees of the British Museum. Shared under a creative commons attribution-non-commercial-ShareAlike 4.0 International (CC BY-NC-SA 4.0) licence.

Whiskers formed a prominent part of the construction of the dandified man in 1810ʹs *A Modern Stride of Barber-ism* (Figure 2). Here, the nattily dressed figure is seen striding away from a corner shop in Bishopsgate Street in London, bearing the legend ‘Cabinet of Caput Coverings’, over a window in which can be seen male and female busts wearing wigs and hairpieces. The joke rests on the implication that the man’s elaborate and styled whiskers, like the barber’s constructions in the window, are an effete and unnatural adornment, reinforced by the word play on ‘barbarism’. The use of the term ‘caput’ also locates the whiskers as a foreign, German affectation.^87^^87^Anon, *A Modern Stride of Barber-ism* (London, 1810).

Similar tropes could be found elsewhere. The author of ‘Singular Fashions’ in the *New British Ladies Magazine* of 1818 complained about ‘dandies who display their tight-laced bodies and Cossack crops in the purlieus of St James’s [and] the whiskers which adorn their countenance’.^88^^88^‘Singular fashions’, *The New British Ladies Magazine*, 12 August 1818, 68. In a satirical letter to the *Weekly Entertainer* in 1819, ‘Telephus’ feared being mistaken for a dandy. His ‘vigorous whisker’ was, itself, ‘almost enough to ruin me in the estimation of all sober, smooth-faced people’.^89^^89^‘Telephus’ and ‘Dandyism’, *The Weekly Entertainer*, 12 April 1819, 291–92. Theodore Lane neatly captures these attitudes in a satirical print of 1824, titled *The Rival Whiskers* (Figure 4). Here a variety of styles can be seen, both with and without moustaches, but also among different groups of men. To the left of the image, two soldiers are depicted, one with a moustache and side-whiskers, the other with a full set of ‘chin curtain’ whiskers but no moustache. On the right, two elaborately dressed gentlemen parade the pavement, displaying large sets of bushy whiskers. The effect is amplified by the men turning their faces upwards, to show off their magnificent facial hair. Much about the image suggests the effeminate connotations of whiskers. The two men are not only ostentatiously dressed but walk arm in arm. There are also various animalistic allusions in the image, including a number of bees heading for the whiskers of one of the men, references to lions’ manes, and also an advertisement for ‘bears’ grease’ to promote whiskers, suggesting the artificiality of the trend. The caption of the image is also telling. ‘They look not like the Inhabitants o’the Earth, and yet are on’t’. Marked out by their whiskers, these dandyish men are almost literally viewed as alien, in contrast to the proper place of the whiskers on the soldiers’ faces.

**Figure 3.**
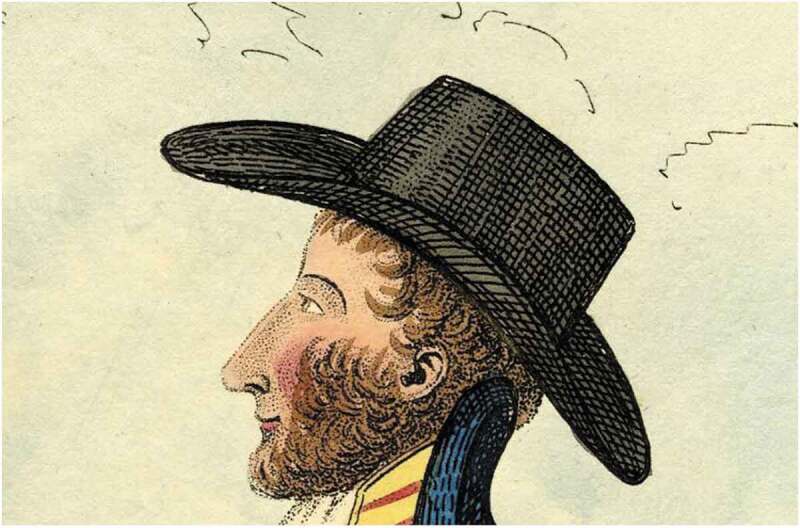
Detail from *A Modern Stride of Barber-ism* (1810). *©*The Trustees of the British Museum. Shared under a creative commons attribution-non-commercial-ShareAlike 4.0 International (CC BY-NC-SA 4.0) licence.

**Figure 4.**
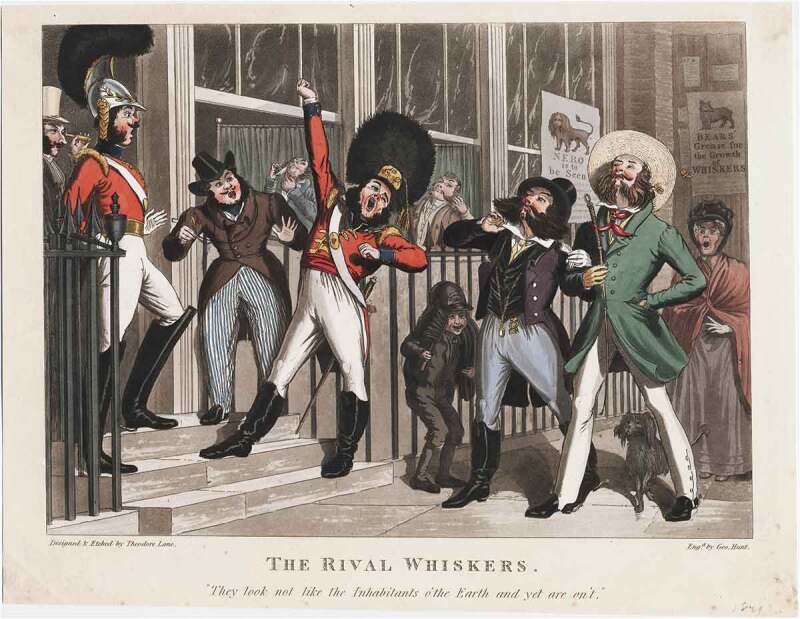
Theodore Lane, *The Rival Whiskers, Designed and Etched by Theodore Lane; Engraved by George Hunt* (London: 1824). Image courtesy of Lewis Walpole Library, Yale University.

Matters were perhaps further complicated by the emergence of a new market for products to imitate, promote, style and colour whiskers, which further blurred the boundaries of acceptable male practices. The early modern period had stigmatised both over-attention towards appearance and the use of cosmetic products by men. From the later eighteenth century, though, came a new market for shaving products, actively encouraging men to use soaps, oils, powders and pastes. As I have argued elsewhere, the use of scent, as well as tropes of softness, ease and luxury in advertisements, complicated the sometimes more austere versions of late Georgian manliness.^90^^90^Withey, *Concerning Beards*, *op. cit*., 233–34. In the early nineteenth century, a range of products emerged specifically targeted at men who either had or wanted whiskers. Wig makers, for example, used the elastic properties of cast steel to contrive false whiskers for men who were unable to grow their own. In 1802, the London perukemaker Robinson of Portman Square began to advertise his ‘Natural Spring Wigs’, which were available ‘with or without whiskers’. Sometimes these were full wigs with false whiskers added, in the colour of the wearer’s choice, which used a system of springs to adhere to the head.^91^^91^For example, see ‘To gentlemen who are desirous of having a natural spring wig’, *Morning Post and Gazetteer*, 20 June 1801, 1. Another London wig maker, Alexander Ross, sold ‘whisker wigs’, which he claimed were extremely popular. Ross urged the public to buy direct from him, since the ‘great demand’ for them rendered him unable to supply to trade.^92^^92^‘Unparalleled improvements’, *Morning Post*, 7 January 1801, 1. The point of the device was again to allow those whom nature had not seen fit to endow with a set of bushy whiskers to participate in what was clearly an emerging and socially important fashion.

Perhaps the main group of cosmetic products were those for promoting growth and also colouring head and facial hair.^93^^93^For the broader growth and symbolism of hair colouring products in this period see J. Strachan, *Advertising and Satirical Culture in the Romantic Period* (Cambridge, 2007), ch. 5; S. MCNamara, ‘Production and practice: hair harvest, hairpieces and hairwork’ in S. Heaton (ed.), *A Cultural History of Hair in the Age of Empire* (London, 2021), 69–79. Products such as ‘Russia Oil’ claimed to make hair ‘grow thick and long, even in bald places, whiskers, eye-brows &c’.^94^^94^Advertisement, ‘Sold by Harmer and Green’, *The Ipswich Journal*, 22 March 1806. In 1807, perfumer John Chasson of Cornhill, London, advertised his ‘Incomparable Fluid’, for changing hair, whiskers and eyebrows from grey or ‘red’ to ‘beautiful and natural shades of brown and black’.^95^^95^Advertisement, ‘A Most Important Discovery’, *The Morning Post*, 20 March 1807. Other products included ‘Day’s Original Hair Water’, to colour ‘Red or Grey Hair, eye-brows, whiskers &c’ – *The Morning Post*, 2 October 1807. Grey whiskers were problematic and ambiguous. In one respect they could be a visual shorthand for the wisdom and maturity associated with long existence. Equally, however, they could suggest the decrepitude and decline of old age. They were also sometimes viewed as an unwelcome side effect of constant wetting when shaving.^96^^96^This was an argument made in an advertisement for ‘Prince’s Russia Oil’, *Oxford and City Herald*, 27 August 1808, 1. The fact that such advertisements specifically targeted men with greying hair suggests that the fashion had also moved beyond younger men. ‘Red’ or ginger whiskers potentially carried negative racial associations with Irish, Scottish or sometimes Jewish ethnicity.^97^^97^Withey, *Concerning Beards*, *op. cit*., 131–33. By 1815, the number of such preparations had proliferated. Indeed, the new market for cosmetic dyeing products highlights perhaps the most unusual manifestation of the trend for whiskers, which was its imitation by women, providing further potential evidence of the extent of the fashion. In advertisements, for example, perfumer John Chasson’s ‘Incomparable Fluid’ was recommended to ‘women of distinction’.^98^^98^‘To the Ladies’, *The Morning Post*, 3 February 1810, 1. At first this apparent printing error was lampooned in an article titled ‘Whiskered Ladies!’ in *The Satirist* magazine, which questioned whether Chasson’s customers would include ‘the Belles of Cockermouth’, or the ‘The Countess Dowager of B – s whiskers’ which were apparently ‘already in great forwardness’.^99^^99^‘Barbara Beardless’ and ‘Whiskered Ladies!’, *The Satirist or Monthly Meteor*, Volume III (London, 1808) 243–45. Chasson was not the only perfumer offering products seemingly for ladies’ whiskers. Another product, the ‘Original Preparation’, advertised ‘To the Ladies’, similarly offered to change ‘red or grey hair, whiskers or eye-brows’ to a ‘beautiful natural colour’.^100^^100^Advertisement, ‘To the Ladies’, *The Morning Post*, 31 October 1807. This could simply have referred to the neutralising of unsightly dark hairs on women’s faces. However, another advertisement by Chasson in fact suggested that some women took to using pencils to draw whiskers on their cheeks. In July 1807, an advertisement for the ‘Tricosian Fluid’ (under the headline ‘Ladies Who Are Desirous of Appearing in Their Native Tresses’) promised to restore hair to its natural colour, while also bypassing the ‘present troublesome mode of pencilling the eyebrows *and whiskers*’ [my emphasis].^101^^101^Advertisement, ‘Ladies who are desirous of appearing in their native tresses’, *The Morning Post*, 1 July 1807. Nowhere in the advertisement were men mentioned.

Such products, together with the sexual ambiguities already carried by whiskers, served to create tensions in terms of gender boundaries, and what stood for acceptable manly appearance and practices. On the one hand whiskers could represent a sub-section of masculinity based on youth, rakishness and a unique style; on the other, however, the emphasis on grooming, and particularly the use and perhaps even sharing of ‘unisex’ cosmetic products, raised the spectre of effeminacy.

## Conclusion

As this article has shown, the emergence of a fashion for whiskers in the early decades of the nineteenth century occurred amid a series of changes to broad concepts of the male body, influenced by key factors including ambiguous and overlapping attitudes related to martial manliness, popular xenophobia and fears about the ‘foreign’, as well as concerns about the physical and moral enervation of young British men. Despite their obvious popularity – at least in some sections of society – whiskers, and their wearers, were ‘othered’.

The arguments against facial hair both reflect and confirm the flux in concepts of masculinity as highlighted by Tosh, Begiato and others. They reveal much about contemporary concerns of how to articulate a manly body that was intrinsically ‘British’. Whiskers supported a range of connotations and contradictions. They could imply martial prowess, or equally an unwelcome symbol of ‘foreign’ revolutionary spirit. They could convey a fashionable body, but also one that could be construed as ‘dandified’, effete or, worse for contemporaries, French! In many respects, whiskers were a distinguishing mark in a society in which the conditions were not yet favourable for the return of facial hair. What stood for positive characteristics and associations in the 1850s did not necessarily do so in the early 1800s. While emulation of the martial body was held to be a strong component of mid-Victorian moustache- and beard-wearing, in the early part of the century it was more muted. By 1828, the author of an article titled ‘Beards and whiskers’ felt confident in asserting the ‘fallacy of the prevailing notion, that beards and whiskers are any evidence of the courage and manhood of the wearer’.^102^^102^Ephorus, ‘Beards and whiskers’, *The Kaleidoscope*, 24 June 1828, 425.

Perhaps even more important was the strength of associations with facial hair as a ‘foreign’ characteristic in the early nineteenth century. Whiskers, moustaches and beards bore unfavourable, indeed xenophobic, connections with continental stereotypes. Claims made in support of beards after 1850 often made a virtue of the ‘Britishness’ (or, more specifically, ‘Englishness’) of the beard, claiming it as an example of national pride that Englishmen could sport such magnificent outcrops of beard. Facial hair in the early century was, however, at least initially, regarded as distinctly un-English. The controversy surrounding the Sepoy mutiny is a case in point. In attempting to deprive the Muslim and Hindu soldiers of their sacred beards, the British commanders had touched a sensitive nerve, and also raised a crucial point of difference. The controversy surrounded the removal of a cultural and religious marker. Indian men were *expected* to wear facial hair. The arguments made in their defence, however, did not extend to advocating that British troops should follow suit and grow their own.

Finally, the fashion for whiskers also sheds new light on the issue of gender and what stood for ‘acceptable’ male behaviours and appearance. For many, whiskers embodied an effete masculinity, one linked to dandyism and young, fey elites. While there is some evidence that they were worn by men lower down the social scale as well, it is impossible to know whether this reflected a broader, national trend, or instead the personal decisions of individual men. References to criminals and runaways with whiskers, for example, may reflect their usefulness as a prosthetic, which could be grown or removed as a disguise. Literally and metaphorically, though, whiskers blurred the boundaries between male and female. The need to style and maintain them encouraged grooming practices and products that some were still uncomfortable with. Nevertheless, the fact that a market for them emerged is suggestive of the popularity of the trend.

Recovering the contexts within which a previously overlooked facial hair fashion in the early nineteenth century was understood, and the debates it engendered, therefore offers a new and novel perspective upon embodied manliness, and demonstrates the importance of facial hair as a marker of masculinity through time. It also provides a useful reminder of the dangers of assuming that fashion and male appearance were either normative or hegemonic.

